# Ultrafast magnetization modulation induced by the electric field component of a terahertz pulse in a ferromagnetic-semiconductor thin film

**DOI:** 10.1038/s41598-018-25266-2

**Published:** 2018-05-02

**Authors:** Tomoaki Ishii, Hiromichi Yamakawa, Toshiki Kanaki, Tatsuya Miyamoto, Noriaki Kida, Hiroshi Okamoto, Masaaki Tanaka, Shinobu Ohya

**Affiliations:** 1 Department of Electrical Engineering and Information Systems, The University of Tokyo, 7-3-1 Hongo, Bunkyo-ku, Tokyo, 113-8656 Japan; 2 Department of Advanced Materials Science, Graduate School of Frontier Sciences, The University of Tokyo, Chiba, 277-8561 Japan; 3 Center for Spintronics Research Network, Graduate School of Engineering, The University of Tokyo, 7-3-1 Hongo, Bunkyo-ku, Tokyo, 113-8656 Japan; 4 Institute of Engineering Innovation, Graduate School of Engineering, The University of Tokyo, 7-3-1 Hongo, Bunkyo-ku, Tokyo, 113-8656 Japan

## Abstract

High-speed magnetization control of ferromagnetic films using light pulses is attracting considerable attention and is increasingly important for the development of spintronic devices. Irradiation with a nearly monocyclic terahertz pulse, which can induce strong electromagnetic fields in ferromagnetic films within an extremely short time of less than ~1 ps, is promising for damping-free high-speed coherent control of the magnetization. Here, we successfully observe a terahertz response in a ferromagnetic-semiconductor thin film. In addition, we find that a similar terahertz response is observed even in a *non-magnetic* semiconductor and reveal that the electric-field component of the terahertz pulse plays a crucial role in the magnetization response through the spin-carrier interactions in a ferromagnetic-semiconductor thin film. Our findings will provide new guidelines for designing materials suitable for ultrafast magnetization reversal.

## Introduction

In ferromagnetic materials, typically more than a few hundred picoseconds are necessary to reverse the magnetization when using spin-transfer torque or light irradiation^1–13^, limiting the operational speed of magnetic memory devices. Meanwhile, using a terahertz light pulse, a strong electromagnetic field can be induced within an extremely short time of less than ~1 picosecond in ferromagnetic thin films, in which the spin-lattice relaxation is too slow to follow the electromagnetic fields^11^. Thus, terahertz pulse control of the magnetization is promising for ultrafast magnetization reversal within a few picoseconds. In the previous studies on terahertz pump-probe measurements, a tiny modulation in the magnetization by a terahertz pulse was demonstrated for ferromagnetic-metal thin films such as Co, Ni, Fe and CoFeB^11–13^. Until now, the origin of this phenomenon has been attributed to the Landau-Lifschitz-Gilbert (LLG) torque^11–13^, which is induced by the magnetic-field component of the terahertz pulse, and to the demagnetization caused by the heating^11,13^.

Recently, static-electric-field control of the magnetization vector^14–16^, Curie temperature and coercivity^17,18^ has been reported for magnetic metal thin films; however, thus far, the electric-field component of the terahertz pulse has not often been associated with the magnetization modulation^13^. In non-magnetic materials, optical properties are known to be influenced by the modulation of the spatial carrier distribution induced by the electric field of light^19–21^, which is called the Franz-Keldysh effect (FKE). The FKE has been investigated mainly for semiconductors rather than metals because semiconductors have a low carrier density and are sensitive to electric fields. GaAs is a suitable semiconductor for our study because it becomes ferromagnetic when doped with Mn and because Mn-doped GaAs (GaMnAs) is fairly sensitive to an external optical stimulus^5,22^. Comparing GaAs samples doped with non-magnetic atoms and with Mn atoms can demonstrate how the electric field of the terahertz pulse influences the magnetization in ferromagnetic GaMnAs.

## Results

### Samples and experimental setup

**Figure 1:**
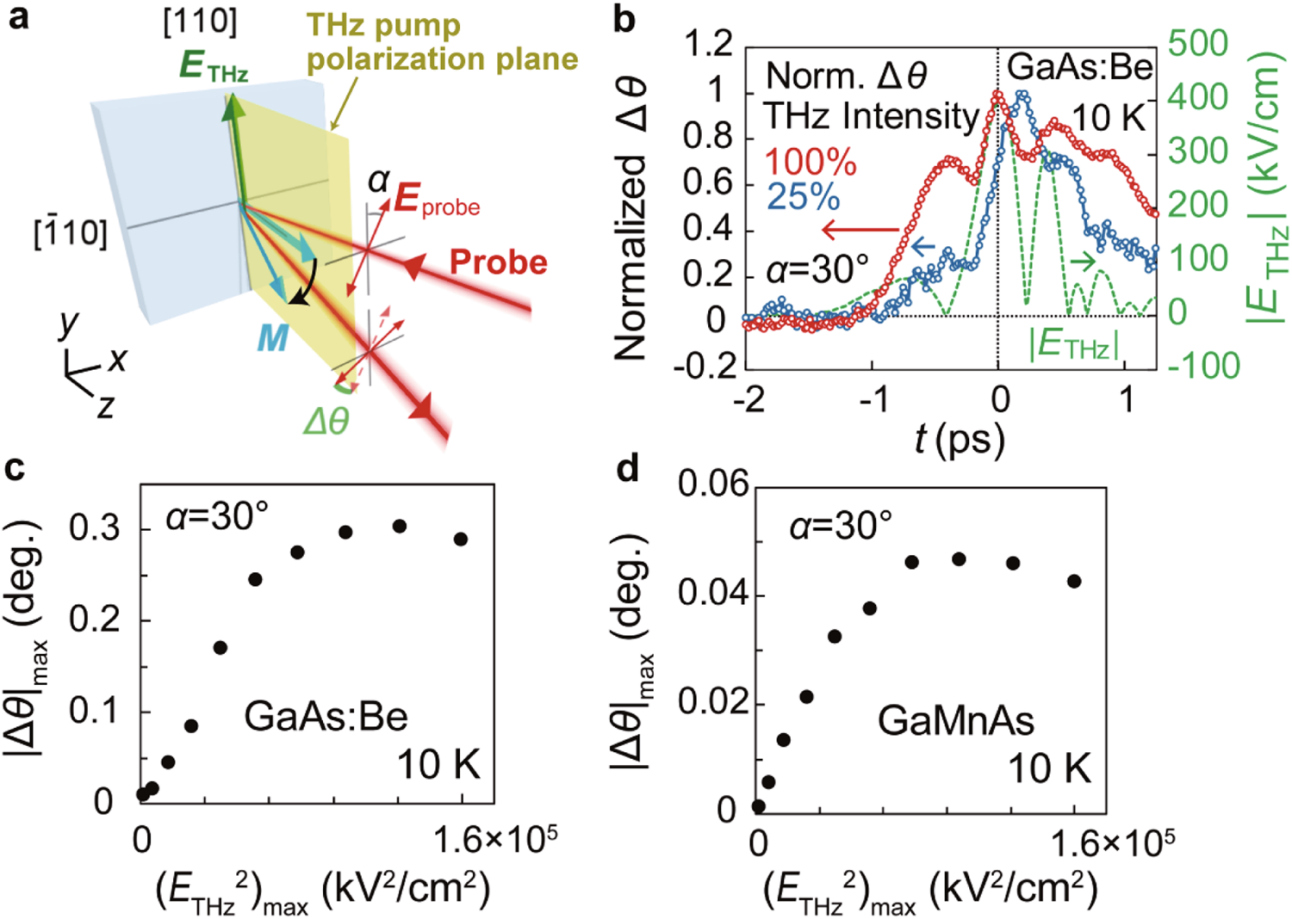
Overview of the experiment and the observation of Δ*θ* induced by the Franz-Keldysh effect. (**a**) Schematic illustration of the experimental setup. The terahertz pump pulse (yellow) is focused on the GaAs:Be or GaMnAs sample surface, and the following probe pulse with a delay time *t* detects the excited dynamics. Both the pump and probe pulses are linearly polarized. The strong terahertz-pump pulse with a centred frequency of 1 THz, whose electric field *E*_THz_ is along the [110] axis, is generated by optical rectification using a LiNbO_3_ crystal. The incident angle of the probe pulse is tilted by 10° from the sample normal towards the in-plane [110] axis. The magnetization (pale blue arrow) is tilted from the sample normal by the terahertz pump pulse. The angle of the probe polarization plane from the [110] axis towards the $[1\bar{1}0]\,$ axis is defined as *α*. (**b**) Time evolution of Δ*θ* (red circles) measured at 10 K without an external magnetic field for GaAs:Be when applying *E*_THz_ expressed by the green dotted curve. Δ*θ* is normalized by its maximum value. The blue circles express Δ*θ* when the intensity *E*_THz_^2^ of the terahertz pump pulse is 25% of the green dotted curve (*i.e*., the maximum *E*_THz_ is 200 kV/cm). *α* is set to 30°. (**c**,**d**) Maximum value of the time evolution of |Δ*θ*| obtained for GaAs:Be (**c**) and GaMnAs (**d**) plotted as a function of the maximum value of *E*_THz_^2^ at 10 K. These measurements are carried out without an external magnetic field when *α* is set to 30°.

In our terahertz pump-probe measurements, we used thin films so that the terahertz pump beam can penetrate them. We use a non-magnetic semiconductor Be-doped GaAs thin film (referred to as GaAs:Be, Be acceptor concentration: 10^19^ cm^−3^, thickness: 40 nm) and a ferromagnetic semiconductor Ga_0.94_Mn_0.06_As thin film (thickness: 20 nm) with a perpendicular easy magnetization axis (see Methods). We utilize strong terahertz-pump pulses (~400 kV/cm) with the centered frequency of 1 THz, whose electric field *E*_THz_ is aligned along the [110] axis in the film plane (Fig. 1a). We detect the change Δ*θ* in the polarization rotation of the reflected probe pulse with a delay time *t* relative to the terahertz-pump pulse. All measurements are carried out at 10 K (see Methods).

### Observation of Δ*θ* induced by the FKE

Here, we show that the strong FKE is induced by the terahertz pump pulses both in GaAs:Be and GaMnAs. The FKE induces magnetization modulation and birefringence. In the following discussion, the subscript “max” refers to the maximum value of the transient. In GaAs:Be, evidence of the FKE can be seen in the (*E*_THz_^2^)_max_ dependence of (−Δ*R*/*R*)_max_ and in the time evolution of −Δ*R*/*R* (Supplementary Information Fig. S1); (−Δ*R*/*R*)_max_ increases and saturates with increasing (*E*_THz_^2^)_max_ and the peak positions of −Δ*R*/*R* depend on (*E*_THz_^2^)_max_ (see Supplementary Information Sec. A). These are distinctive features of the FKE^23^. Owing to the polarization of the terahertz pulse along [110], the FKE induces the anisotropy of the reflectivities between [110] and $[1\bar{1}0]\,$ polarized light beams^24^. This anisotropy leads to the birefringence and thus the polarization rotation. These effects are actually observed in *non-magnetic* GaAs:Be, as shown in Fig. 1b, where the angle *α* between the electric field vector *E*_probe_ of the probe and *E*_THz_ (//[110]) is set to 30° (Fig. 1a). As shown in Fig. 1c, |Δθ|_max_ tends to saturate as (*E*_THz_^2^)_max_ increases, confirming that the observed Δ*θ* is induced by the FKE^23^. For GaMnAs, we see that similar saturating behaviour appears in the (*E*_THz_^2^)_max_ dependence of |Δ*θ*|_max_ (Fig. 1d). Here, *α* is also set to 30°. This result indicates that Δ*θ* is mainly attributed to the birefringence due to the FKE. The Δ*θ* value observed for GaMnAs is smaller than that for GaAs:Be because the carrier density of GaMnAs is larger than that of GaAs:Be. Note that the small drop of |Δ*θ*|_max_ at high electric fields is due to the shift of the absorption edge by the FKE^25^. Because the signal induced by the birefringence is not directly related to the magnetization dynamics, this component should be removed.

### Coherent magnetization modulation by *E*_THZ_

**Figure 2:**
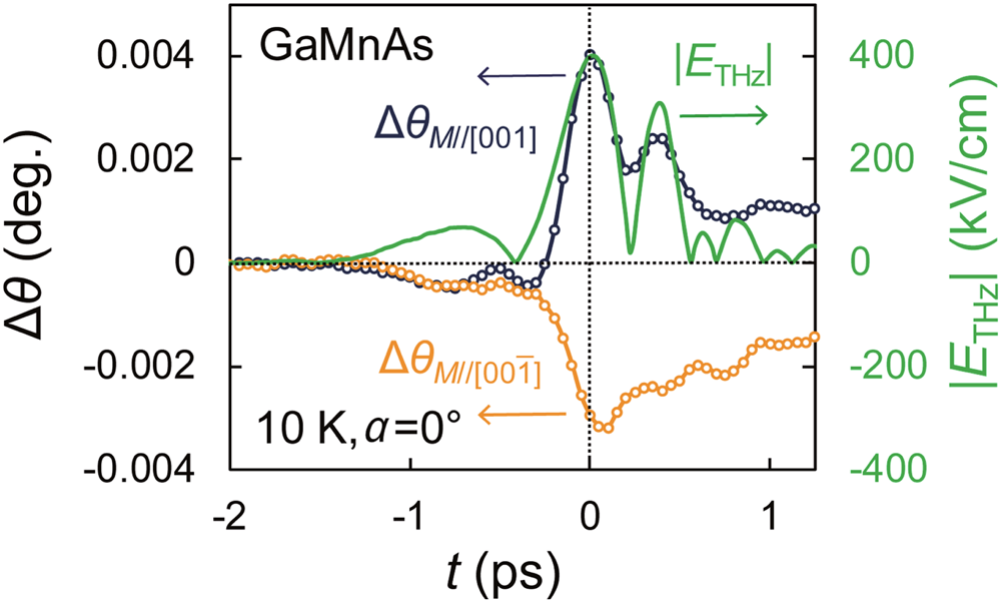
Terahertz response of the GaMnAs film. Blue and orange open circles represent Δ*θ* measured at 10 K for the Ga_0.94_Mn_0.06_As thin film when *E*_probe_//*E*_THz_ (*α* = 0°) with the magnetic field of 30 mT is applied in the [001] and $[00\bar{1}]$ directions, respectively. |*E*_THz_| is shown by the green solid curve.

By setting *E*_probe_//*E*_THz_(//[110]) (*i.e., α* = 0°), the effect of the birefringence can be minimized, and the magnetic signal becomes dominant (Supplementary Information Sec. B). In fact, the sign of Δ*θ* is almost perfectly inverted when the magnetic field (=30 mT), which is applied perpendicular to the sample surface to align the magnetization, is reversed (orange and blue circles in Fig. 2). This means that the observed signal in Fig. 2 is purely a magnetic signal and is almost proportional to the change Δ*M*_perp_ in the perpendicular magnetization *M*_perp_, *i.e*., Δ*θ* is mainly attributed to the polarization rotation Δ*θ*_MOKE_ induced by the magneto-optical Kerr effect. Note that the terahertz modulation of the magnetization is not caused by the magnetic field of the terahertz pulse, because the observed Δ*θ* is three orders of magnitude larger than that calculated by the LLG-torque model (Supplementary Information Sec. D); rather, it is explained by the electric field of the terahertz pulse, *i.e*., the FKE. As shown below, in addition to this component, Δ*θ* incorporates a small polarization rotation Δ*θ*_bir_ induced by the birefringence, which remains owing to the small deviation from the ideal alignment of *E*_probe_//*E*_THz_, and a component of the magnetic linear dichroism (Δ*θ*_MLD_), which is proportional to the square of the in-plane magnetization Δ*M*_in_^26^.

To quantitatively understand the magnetization dynamics, we perform the analysis using the dielectric tensor^27^,

1 $$\tilde{\varepsilon }=(-\begin{array}{ccc}{\varepsilon }_{xx} & {\varepsilon }_{xy} & 0\\ {\varepsilon }_{xy} & {\varepsilon }_{yy} & 0\\ 0 & 0 & {\varepsilon }_{zz}\end{array})$$

**Figure 3:**
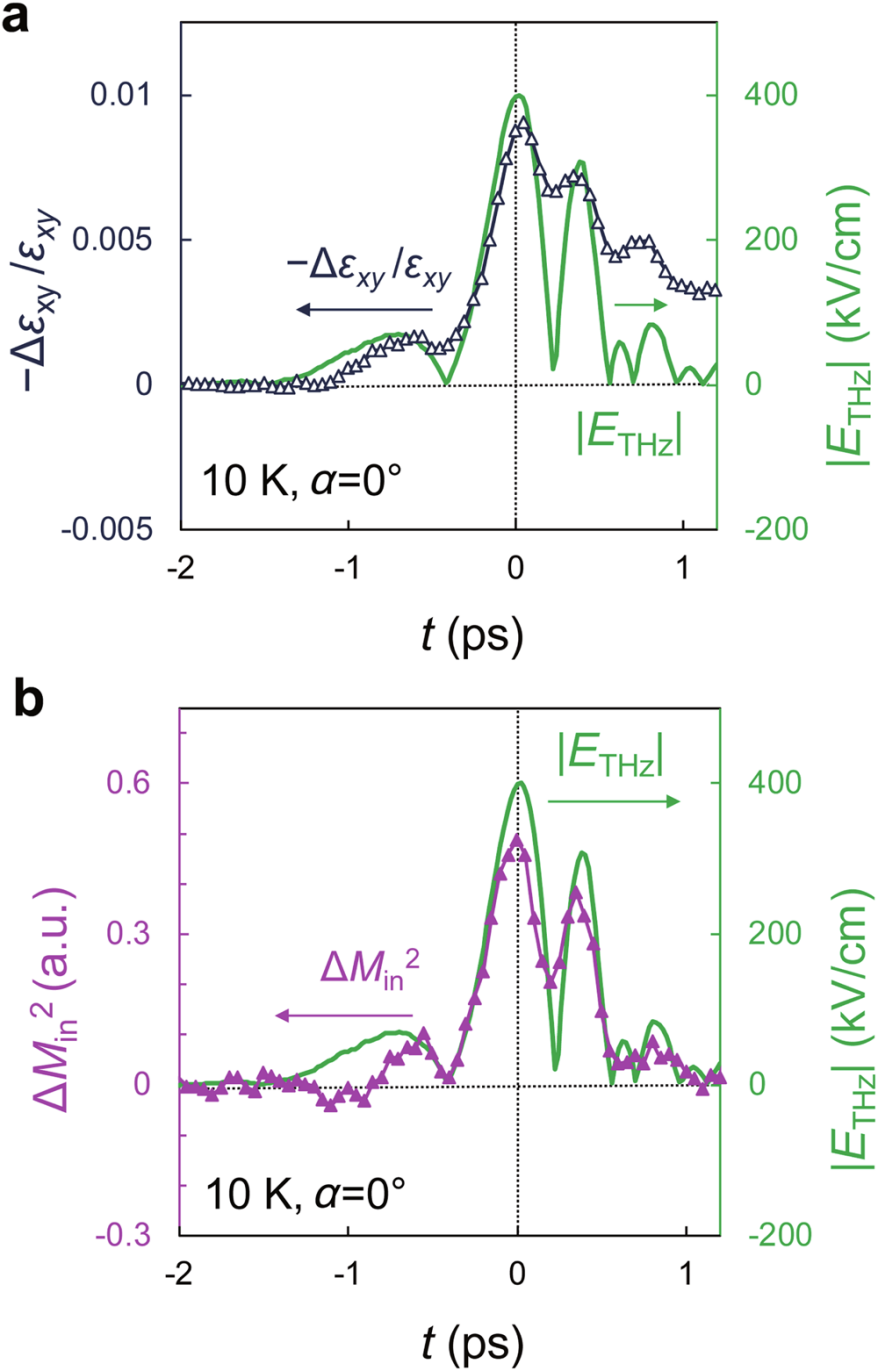
Magnetization modulation by *E*_THz_. (**a,b**) The time evolution of $-\Delta {\varepsilon }_{xy}/{\varepsilon }_{xy}$ (dark blue plot in **a**) and that of Δ*M*_in_^2^ (purple plot in **b**). |*E*_THz_| is shown by the green solid curve. Here, *α* is 0°.

Here, *x*// $[1\bar{1}0]\,$, *y*//[110] and *z*//[001] (Fig. 1a). Using this tensor and the Onsager relations, we can express Δ*θ*_bir_, Δ*θ*_MOKE_ and Δ*θ*_MLD_ using *ε*_*xx*_, *ε*_*yy*_, *ε*_*xy*_ and *ε*_*zz*_, as described in Supplementary Information Sec. B. Δ*θ*_MOKE_, which is proportional to Δ*M*_perp_, is obtained by $({\rm{\Delta }}{\theta }_{M//[001]}-{\rm{\Delta }}{\theta }_{M//[00\bar{1}]})/2$, where ${\rm{\Delta }}{\theta }_{M//[001]}$ and ${\rm{\Delta }}{\theta }_{M//[00\bar{1}]}$ denote Δ*θ* measured with the magnetic field applied in the [001] and $[00\bar{1}]$ directions shown in Fig. 2, respectively. Because Δ*θ*_MOKE_ is influenced by not only the change in *ε*_*xy*_ but also the change in *ε*_*yy*_ (Supplementary Information Sec. B), we derive −Δ*ε*_*xy/*_*ε*_*xy*_ (Fig. 3a), which is purely proportional to Δ*M*_perp_ divided by the initial perpendicular magnetization before the pump pulse irradiation (Supplementary Information Sec. B). Here, Δ*ε*_*xy*_ is the change in *ε*_*xy*_. Figure 3a shows that *M*_perp_ is indeed modulated by up to 1% and that it coherently follows the terahertz electric field.

For coherent magnetization control, the magnetization must tilt following the electric field of the terahertz pulse. We therefore examine the in-plane magnetization response. We derive Δ*θ*_MLD_, which is proportional to Δ*M*_in_^2^, using the relation Δ*θ*_MLD_ = Δ*θ* − Δ*θ*_bir_ − Δ*θ*_MOKE_, where Δ*θ*_bir_ is derived from experimental Δ*R*/*R* (Supplementary Information Sec. B). In Fig. 3b, the dynamics of Δ*M*_in_^2^ (purple plot) show a time evolution similar to that of the perpendicular magnetization dynamics (dark blue plot in Fig. 3a), *i.e*., Δ*M*_in_^2^ increases when *M*_perp_ decreases (or when −Δ*ε*_*xy*_/*ε*_*xy*_ increases). The geometrical calculation demonstrates that −Δ*M*_perp_ is proportional to Δ*M*_in_^2^ (Supplementary Information Sec. C). Therefore, our results indicate that the magnetization is indeed tilted by the terahertz pulse. These results clearly indicate that the magnetization coherently follows the ultrafast oscillation of the electric field of the terahertz pulse via the FKE.

## Discussion

**Figure 4:**
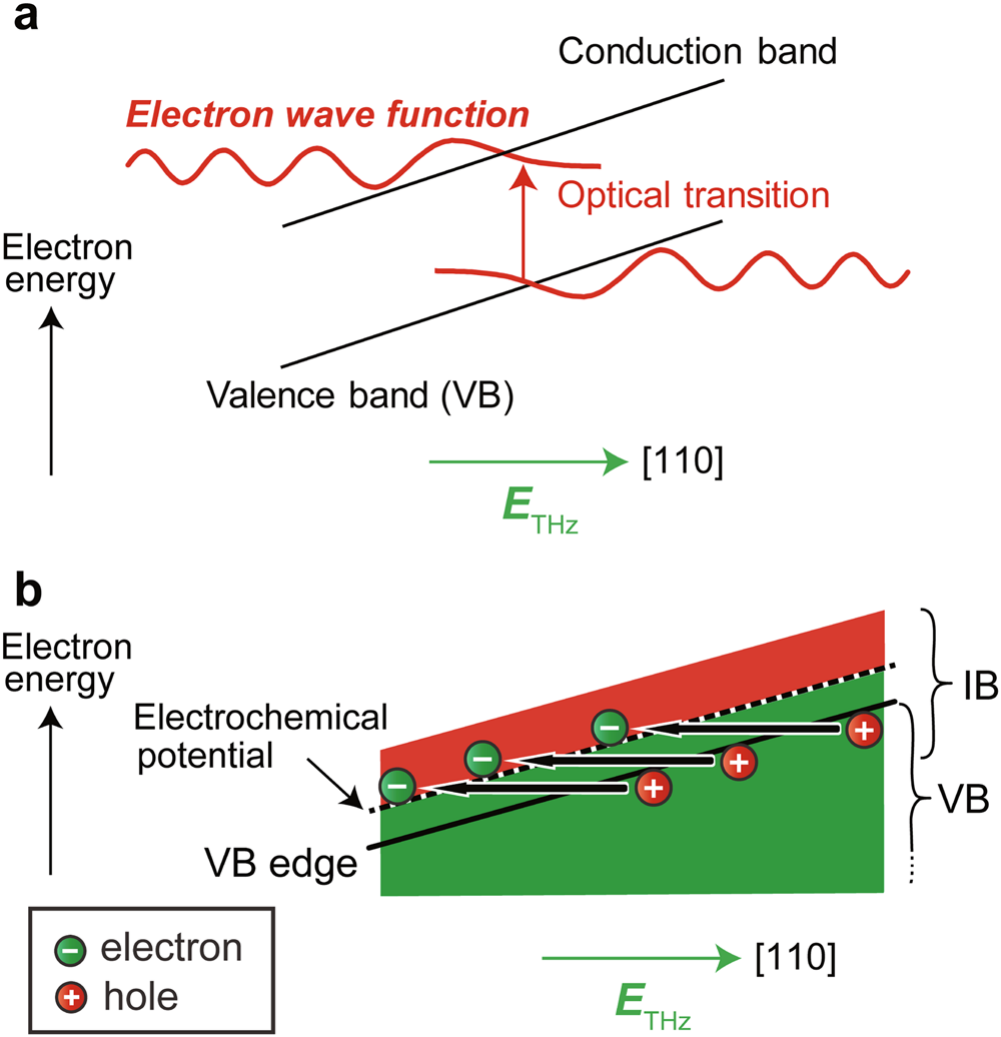
Magnetization modulation via the modulation of the spatial carrier distribution induced by the electric field of light. (**a**) Modulation of the band structure by the FKE. The band structure is spatially tilted by *E*_THz_, enabling optical transitions with an energy smaller than the band gap. The red curves represent the electron wave functions. (**b**) Spatial band diagram of GaMnAs while *E*_THz_ is applied. The green and red regions are filled by electrons and holes, respectively. The IB and the VB overlap. Owing to the electric field, electrons in the VB can move to the IB.

We now discuss the physical mechanism of the magnetization modulation by the terahertz pulse. In the FKE, the anisotropy of the refractive index is induced by the anisotropic modulation of the band structure; owing to the application of a terahertz electric field along [110], the band structure is spatially tilted along [110] (Fig. 4a). This enables optical transitions with energies smaller than the band gap for [110]-polarized light, thus modulating the reflectivity of the material. In GaMnAs, while *E*_THz_ is applied, not only the valence band (VB) but also the electrochemical potential (EP) is tilted because carriers cannot diffuse within a few picoseconds (Fig. 4b). However, electrons can move between the VB and the impurity band (IB) (Fig. 4b), leading to the modulation in the local carrier concentration in the IB. Because ferromagnetism is induced by the double-exchange interaction between the IB holes in GaMnAs, the modulation in the carrier density of the IB induces the modulation of the magnetization^28,29^ and its direction via spin-orbit interaction^5^ or the inducement of orbital angular momentum^30^.

At first glance, the electric field of the terahertz pulse appears to be not related to the magnetization; however, our results strongly suggest that it plays a crucial role in the terahertz response of the magnetization via the FKE. This new mechanism, *the magnetization modulation via the modulation of the spatial carrier distribution induced by the electric field of light*, will provide guidelines for designing materials that are suitable for coherent control of the magnetization using terahertz pulses and will provide a new approach to the ultrafast magnetization reversal.

## Methods

### Samples

The GaMnAs sample consists of (from top to bottom) Ga_0,94_Mn_0.06_As (20 nm)/In_0.2_Al_0.8_As (500 nm)/GaAs (100 nm), which was grown on a semi-insulating GaAs (001) substrate via low-temperature molecular beam epitaxy. After growth, this sample was annealed at 180 °C for 68 h. The Curie temperature of the film is 125 K. The GaMnAs film has a coercivity of 15 mT at 10 K. The GaAs:Be sample consists of GaAs:Be (Be: 10^19^ cm^−3^, 40 nm)/In_0.2_Al_0.8_As (500 nm)/GaAs (100 nm) grown on a semi-insulating GaAs (001) substrate.

### Terahertz-pump probe measurements

The terahertz-pump probe measurements were performed using a pulsed-light source with a repetition rate of 1 kHz. Both pump and probe pulses were linearly polarized. The strong terahertz-pump pulse with a centred frequency of 1 THz, whose electric field *E*_THz_ was aligned along [110] in the film plane (Fig. 1a), was generated by tilted-pulse-front optical rectification in a LiNbO_3_ crystal with a tilted-pump-pulse-front scheme^31,32^. The measurement of *E*_THz_ is described in detail in ref.^33^. The terahertz intensity was adjusted by rotating two wire-grid polarizers. The time duration of the probe pulse was 90 fs, and the wavelength was 800 nm. The delay time of the probe pulse relative to the pump pulse was controlled by changing the length of the optical path of the probe pulse. Δ*θ* was detected by a balanced detection technique using a half-wave plate, a polarizing beam splitter, a pair of balanced silicon photodiodes and a boxcar integrator. We defined the time origin (*t* = 0 ps) of the terahertz pulse as the time when the terahertz electric field reaches a maximum. All measurements are carried out at 10 K.

## Electronic supplementary material

Supplementary Information
