# Supplementary material for: Ultrafast magnetization modulation induced by the electric field component of a terahertz pulse in a ferromagnetic-semiconductor thin film
Source: Sci Rep. 2018 May 2;8:6901. doi: 10.1038/s41598-018-25266-2 (PMC5931997; doi:10.1038/s41598-018-25266-2)
Supplement: Supplementary file 1 — Supplementary Information [file 41598_2018_25266_MOESM1_ESM.docx]

Supplementary Information

**Ultrafast magnetization modulation induced by the electric field component of a terahertz pulse in a ferromagnetic-semiconductor thin film**

Tomoaki Ishii^1^, Hiromichi Yamakawa^2^, Toshiki Kanaki^1^, Tatsuya Miyamoto^2^, Noriaki Kida^2^, Hiroshi Okamoto^2^, Masaaki Tanaka^1,3^ and Shinobu Ohya^1,3,4^

^1^*Department of Electrical Engineering and Information Systems, The University of Tokyo, 7-3-1 Hongo, Bunkyo-ku, Tokyo 113-8656, Japan*

^2^*Department of Advanced Materials Science, Graduate School of Frontier Sciences, The University of Tokyo, Chiba 277-8561, Japan*

^3^*Center for Spintronics Research Network, Graduate School of Engineering, The University of Tokyo, 7-3-1 Hongo, Bunkyo-ku, Tokyo 113-8656, Japan*

^4^*Institute of Engineering Innovation, Graduate School of Engineering, The University of Tokyo, 7-3-1 Hongo, Bunkyo-ku, Tokyo 113-8656, Japan*

**A. Differential reflectivity of the GaAs:Be sample**

To check that the Franz-Keldysh effect (FKE) occurs in our system, we measured the pump-power dependence of the maximum value of –Δ*R*/*R* in its time evolution [defined as (–Δ*R*/*R*)_max_] (Fig. S1a) for GaAs:Be. Here, Δ*R* is the change in reflectivity *R*. We see that (–Δ*R*/*R*)_max_ increases and then saturates with an increase in the pump power (Fig. S1b). In Fig. S1b, in the high intensity regime (*i.e.* when the maximum terahertz electric field *E*_THz_ is larger than 240 kV/cm), the strong electric field enables the electronic bands of GaAs to adiabatically follow the terahertz electric field and the quasi-static FKE appears. In this case, Δ*R* scales with *E*_THz_. Indeed, in our experimental data (solid circles in Fig. S1b), (–Δ*R*/*R*)_max_ shows a square root dependence on the maximum of *E*_THz_^2^ (see the black solid curve). Meanwhile, (–Δ*R*/*R*)_max_ shows an almost linear dependence on the pump power when the maximum terahertz electric field *E*_THz_ is less than 240 kV/cm (see the dotted line in Fig. S1b), because carriers can dynamically follow the terahertz electric field but the bands cannot adiabatically follow the terahertz electric field in this regime^S1^, which is called the dynamical FKE regime. These are characteristic features of the FKE, which have been well understood in previous studies of the FKE in GaAs using terahertz pump-probe measurements^S^^[[1]](#endnote-1)^. The above-mentioned results are evidence that the FKE actually occurs in the GaAs:Be sample used in our study.

The time evolution of –Δ*R*/*R* (Fig. S1a) can be understood as follows. In the dynamical FKE regime (blue circles in Fig. S1a), the observed peaks (*t* = –0.4, 0.3 ps) of –Δ*R*/*R* are located at the positions of the dips of the |*E*_THz_| – *t* curve (green dotted curve). In this regime, the optical response is governed by the ponderomotive energy^S1^ *U*_p_ (*t*), which is defined as the average kinetic energy of the oscillatory motion of charged particles,

$$U_{p}\left( t \right)=\frac{e^{2}}{2m}\left\langle\bar{\left( \int_{-\infty}^{t} E_{\mathrm{THZ}}\left( t^{'} \right)dt' \right)^{2}} \right\rangle, (S1)$$

where, *e* is the elementary charge, *m* is the mass and $\left\langle\bar{} \right\rangle$ indicates the time average. The integral function of *E*_THz_ indicates that *U*_p_ has peaks at *E*_THz_=0. The expected peak positions are consistent with the observed peak positions (*t* = –0.4, 0.3 ps), as shown by the blue circles in Fig. S1a. Meanwhile, in the quasi-static FKE regime (red circles in Fig. S1a), since the conduction and valence bands of GaAs are modulated by acquiring energy *eE*_THz_*y* (*y//E*_THz_) without any delay, peaks of the optical response (–Δ*R*/*R*) are expected to approach the peak positions of *E*_THz_*.* Indeed, we observed the peaks of –Δ*R*/*R* at *t ~* 0 and 0.5 ps (red circles) are located fairly close to the peaks of |*E*_THz_|–*t* (green dotted curve). This behavior in the time evolution gives another evidence of the FKE.


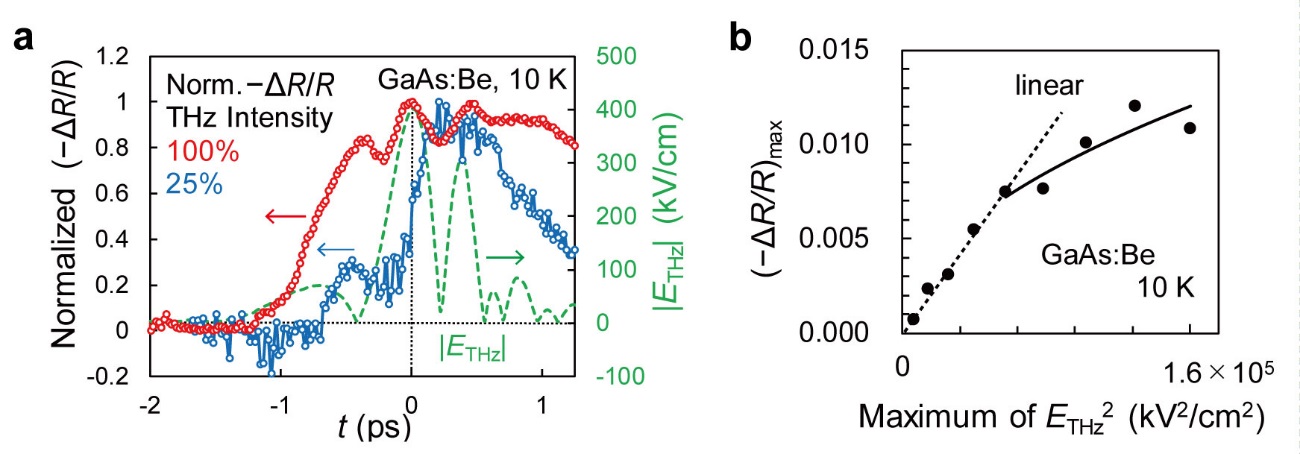


**Figure S1 | Differential reflectivity for the GaAs:Be sample. a,** Red circles show the time evolution of the change (Δ*R*) in the reflectivity (*R*) measured at 10 K for GaAs:Be when the electric field *E*_THz_ of the terahertz pulse expressed by the green dotted curve is applied to the sample. Here, −Δ*R/R* is normalized by its maximum value. Blue solid circles show –Δ*R*/*R* when the intensity *E*_THz_^2^ of the terahertz pump pulse is 25% of the green dotted curve (*i.e.* the maximum *E*_THz_ is 200 kV/cm). The angle between the electric field *E*_probe_ of the probe pulse and *E*_THz_ is set at 30º. **b,** (–Δ*R*/*R*)_max_ obtained for GaAs:Be plotted as a function of *E*_THz_^2^ at 10 K when the angle between *E*_probe_ and *E*_THz_ is 30º.

**B. Derivation of Δ*θ*_MLD_ and Δ*M*_in_**

The polarization rotation *θ* of the probe pulse before the pump irradiation (in-plane magnetization *M*_in_=0) is given by^S^^[[2]](#endnote-2)^

$$\theta\left( t\ll0 \right)=-\frac{\varepsilon_{xy}+s_{1}\sin2\alpha}{\left( 1-\varepsilon_{a} \right)\sqrt{\varepsilon_{a}}}, (S2)$$

where *α* is the angle between the incident probe polarization and the *y* axis (see Fig. S2), and

$$\varepsilon_{a}=\frac{1}{2}\left( \varepsilon_{xx}+\varepsilon_{yy} \right), \varepsilon_{s}=\frac{1}{2}\left( \varepsilon_{xx}-\varepsilon_{yy} \right)\cong s_{1}+s_{2}M_{\mathrm{in}}^{2}, (S3)$$

where *ε_xx_* (*ε_xx_* =12 for GaAs and GaMnAs), *ε_yy_* (=12 for GaAs and GaMnAs) and *ε_xy_* are the elements of the dielectric tensor (see equation (1) in the main text), and *s*_1_ and *s*_2_ are constants. In the last term of equation (S3), from the Onsager equation, *ε_s_* is expanded using a power series of the squared in-plane (*x-y* plane) magnetization *M*_in_^2^. Note that before the terahertz-pump pulse irradiation, *M*_in_^2^=0.

By the terahertz-pump pulse irradiation, the dielectric tensor is modulated; *ε_xy_*, *ε_a_* and *ε_s_* are changed to *ε_xy_*+Δ*ε_xy_*, *ε_a_*+Δ*ε_a_* and *s*_1_+Δ*s*_1­_+ *s*_2_Δ*M*_in_^2^, respectively, where Δ*ε_xy_*, Δ*ε_a_*, Δ*s*_1­_ and Δ*M*_in_ are the changes in *ε_xy_*, *ε_a_*, *s*_1­_ and *M*_in_, respectively. In the following, using Δ*ε_xy_*, Δ*ε_a_*, Δ*s*_1­_ and Δ*M*_in_, we express the change Δ*θ*_MOKE_ in the magneto-optical polar Kerr rotation, the change Δ*θ*_bir_ in the polarization rotation induced by the birefringence and the change (Δ*θ*_MLD_) in the polarization rotation by the magnetic linear dichroism as follows.

$$\Delta\theta_{\mathrm{MOKE}}=-\frac{\varepsilon_{xy}+\Delta\varepsilon_{xy}}{\left( 1-\varepsilon_{a}-{\Delta\varepsilon}_{a} \right)\sqrt{\varepsilon_{a}+\Delta\varepsilon_{a}}}+\frac{\varepsilon_{xy}}{\left( 1-\varepsilon_{a} \right)\sqrt{\varepsilon_{a}}} ,$$

$$\Delta\theta_{\mathrm{bir}}=-\frac{\left( s_{1}+\Delta s_{1} \right)\sin2\alpha}{\left( 1-\varepsilon_{a}-{\Delta\varepsilon}_{a} \right)\sqrt{\varepsilon_{a}+\Delta\varepsilon_{a}}}+\frac{s_{1}\sin2\alpha}{\left( 1-\varepsilon_{a} \right)\sqrt{\varepsilon_{a}}} , (S4)$$

$$\Delta\theta_{\mathrm{MLD}}=-\frac{s_{2}\Delta M_{\mathrm{in}}^{2}\sin2\alpha^{'}}{\left( 1-\varepsilon_{a}-{\Delta\varepsilon}_{a} \right)\sqrt{\varepsilon_{a}+\Delta\varepsilon_{a}}} ,$$

where $\alpha'$ is the angle between the incident probe polarization and the direction of *M*_in_ (Fig. S3). We derived $\Delta M_{\mathrm{in}}^{2}$ from Δ*θ*_MLD_ (the derivation of Δ*θ*_MLD_ is explained below). We cannot determine $\alpha'$ from our experimental results; however, for the analysis of the data measured with *E*_probe_//*E*_THz_, we take $\sin2\alpha'=1$, *i.e.* $\alpha'$= 45° or 225°, which corresponds to a typical easy magnetization axis (*i.e.* one of the <100> axes). The obtained $\Delta M_{\mathrm{in}}^{2}$ is shown in Fig. 3b in the main text. Note that we use arbitrary unit for $\Delta M_{\mathrm{in}}^{2}$.

In our optical alignment of *E*_probe_//*E*_THz_, Δ*θ*_bir_ is still remaining due to the small deviation from the ideal condition *α*=0°. For the analysis of the magnetization dynamics, we have excluded Δ*θ*_bir_ from the observed Δ*θ* as follows. We measured the time evolution of Δ*R*/*R* at 10 K for GaMnAs (Fig. S4a). Using the experimental Δ*R*/*R*, Δ*ε_a_* and Δ*ε_s_* (change in *ε_s_*) are estimated by the following equations,

$$\Delta n \cong-\frac{n^{2}-1}{4}\left( -\frac{\Delta R}{R} \right)\left( 1+\frac{n-1}{2}\left( -\frac{\Delta R}{R} \right) \right)^{-1},$$

$$\Delta\varepsilon_{yy}=\left( n+\Delta n \right)^{2}-\varepsilon_{yy}, n^{2}=\varepsilon_{yy}, (S5)$$

$$\Delta\varepsilon_{a}=\frac{1}{2}\Delta\varepsilon_{yy}, \Delta\varepsilon_{s}=-\frac{1}{2}\Delta\varepsilon_{yy},$$

where Δ*n* is the change in refractive index *n*. Generally, the influence of Δ*M*_in_ on Δ*R* is negligible^S^^[[3]](#endnote-3)^. This is because the higher-order terms such as $s_{2}\Delta M_{\mathrm{in}}^{2}$ are much smaller than the zeroth order term $\Delta s_{1}$ in the Taylor expansion of *ε_s_*. Hence, we can use $\Delta\varepsilon_{s}$=$\Delta s_{1}+s_{2}\Delta M_{\mathrm{in}}^{2}\cong\Delta s_{1}$ when we analyze Δ*R*. Note that in the Δ*θ* measurements, which are much more sensitive to the change in the dielectric tensor, we cannot use this approximation. In fact, Δ*θ* signals due to the term $s_{2}\Delta M_{\mathrm{in}}^{2}$ are observed in GaMnAs^S3^. Therefore, Δ*θ*_bir_ can be approximated by,

$$\Delta\theta_{\mathrm{bir}}=-\frac{\left( s_{1}+\Delta s_{1} \right)\sin2\alpha}{\left( 1-\varepsilon_{a}-{\Delta\varepsilon}_{a} \right)\sqrt{\varepsilon_{a}+\Delta\varepsilon_{a}}}+\frac{s_{1}\sin2\alpha}{\left( 1-\varepsilon_{a} \right)\sqrt{\varepsilon_{a}}}$$

$$\cong-\frac{\left( \varepsilon_{s}+\Delta\varepsilon_{s} \right)\sin2\alpha}{\left( 1-\varepsilon_{a}-{\Delta\varepsilon}_{a} \right)\sqrt{\varepsilon_{a}+\Delta\varepsilon_{a}}}+\frac{\varepsilon_{s}\sin2\alpha}{\left( 1-\varepsilon_{a} \right)\sqrt{\varepsilon_{a}}}. (S6)$$

Using these equations, we can obtain Δ*θ*_bir_ if we can obtain the real value of sin 2*α*. Using this Δ*θ*_bir_, Δ*θ*_MLD_ is obtained by Δ*θ*_sum_ − Δ*θ*_bir_, where Δ*θ*_sum_ = $(\Delta\theta_{M//[001]}+\Delta\theta_{M//[00\bar{1}]})/2$ (violet squares in Fig. S4b). Δ*M*_in_^2^ is derived by equation (S4) using the obtained Δ*θ*_MLD_. Here, to obtain Δ*θ*_bir_, we determined sin 2*α* (=0.044, *i.e.* *α*=2.5°) so that Δ*M*_in_^2^ becomes positive and its minimum value becomes zero. The obtained Δ*θ*_MLD_ and Δ*θ*_bir_ are shown as the blue triangles and the red circles in Fig. S4b, respectively. As a reference, the obtained Δ*θ*_MOKE_ is also shown as the dark blue open circles in Fig. S4b. From Δ*θ*_MLD_ as shown in the blue triangles in Fig. S4b, we derived Δ*M*_in_^2^ (Fig. 3b in the main text) using equations (S4) and (S5).


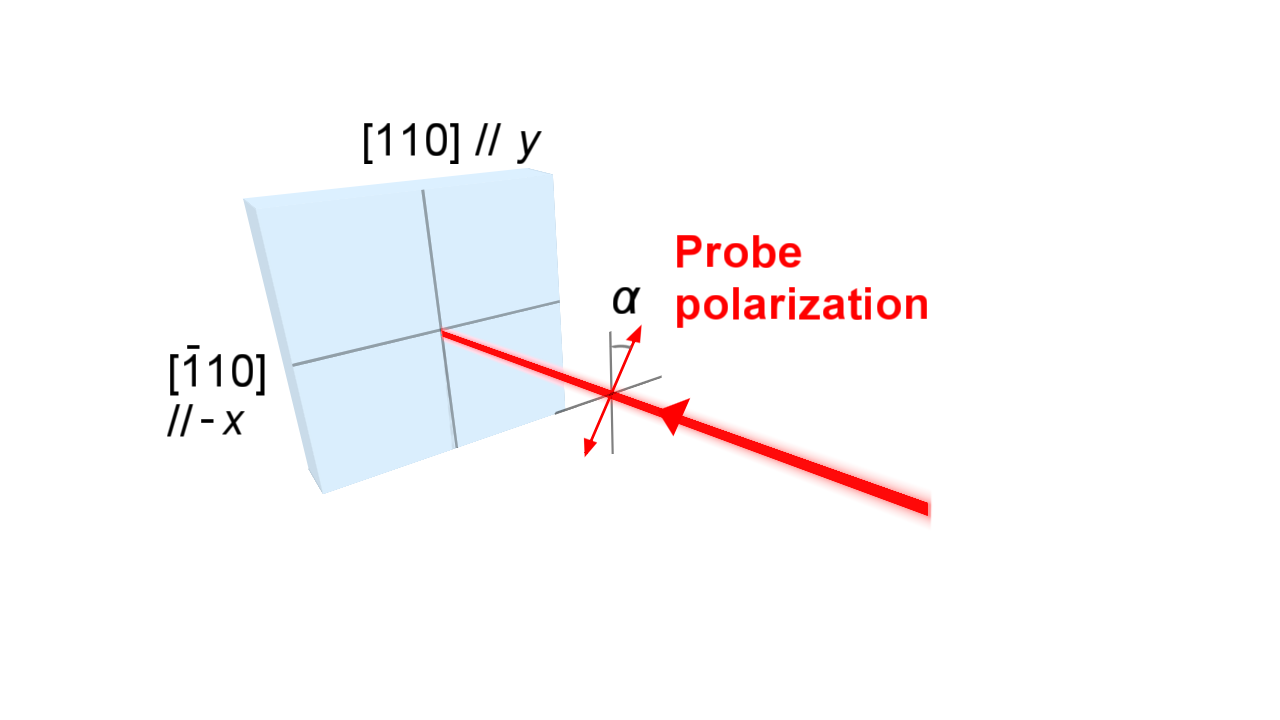


**Figure S2 | Definition of *α*.** *α* is the angle between the incident probe polarization and the *y* axis (//[110]).


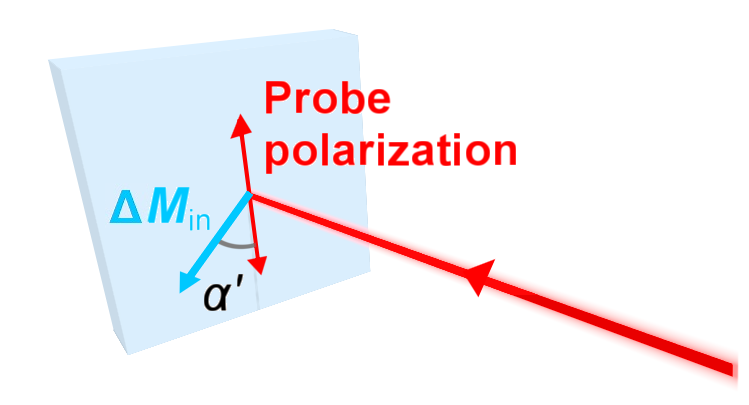


**Figure S3 | Definition of** $\boldsymbol{\alpha}\boldsymbol{'}$**.** $\alpha'$ is the angle between the incident probe polarization and Δ*M*_in_.


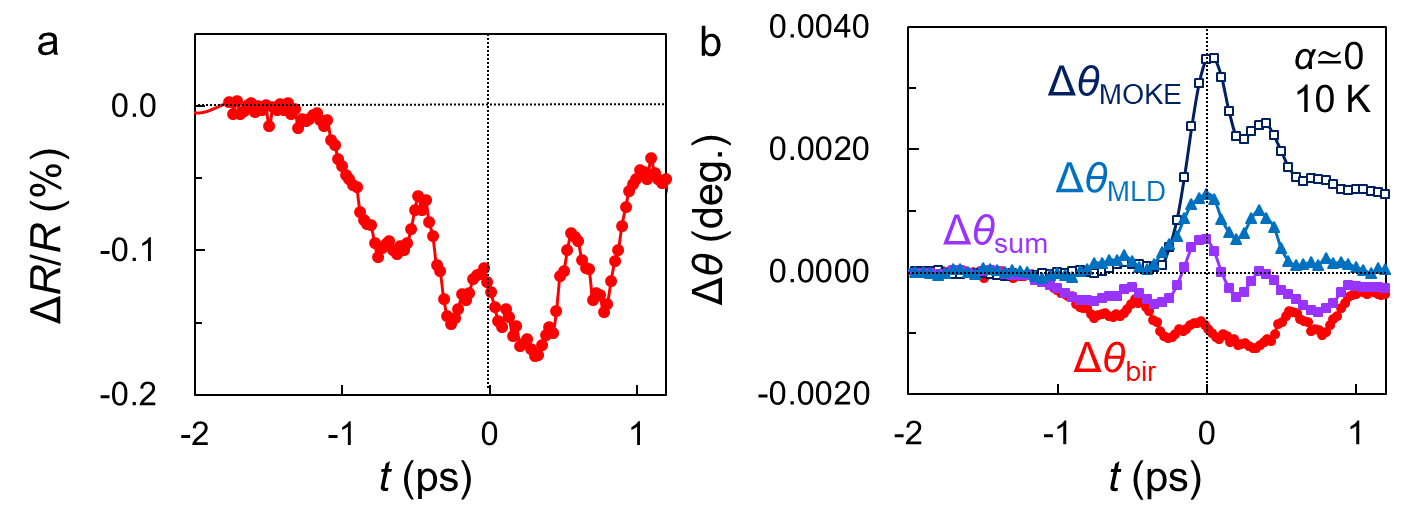


**Figure S4 | Differential reflectivity for GaMnAs.** **a**,Time evolution of the change (Δ*R*) in the reflectivity (*R*) measured at 10 K for GaMnAs when the terahertz pulse is applied to the sample. The angle between *E*_probe_ and *E*_THz_ is 30º. **b**, Time evolution of Δ*θ*_bir_ (red circles) derived from Δ*R*/*R* using sin 2*α*=0.044, that of Δ*θ*_MLD_ (= Δ*θ*_sum_ − Δ*θ*_bir_, blue triangles), that of Δ*θ*_MOKE_ (dark blue open squares) and that of Δ*θ*_sum_ (violet squares), where Δ*θ*_sum_ = $(\Delta\theta_{M//[001]}+\Delta\theta_{M//[00\bar{1}]})/2$.

**C. Relationship between Δ*M*_perp_ and Δ*M*_in_**

When the magnetization direction is tilted by an angle *β* from the initial magnetization direction (//[001], black dotted arrow in Fig. S5), the change Δ*M*_perp_ in perpendicular magnetization *M*_perp_ and Δ*M*_in_ are given by

−Δ*M*_perp_ *=* Δ*M* sin *β*/2,

Δ*M*_in_ *=* Δ*M* cos *β*/2,

where Δ*M* is the change in magnetization *M* (see the red solid arrow in Fig. S5). Δ*M*_in_ is also written by,

Δ*M*_in_ *= M* sin *β*.

Because the tilt angle of *M* is small (*i.e. β*<<1),

−Δ*M*_perp_ $\cong$ Δ*M β*/2*,*

Δ*M*_in_ $\cong$ Δ*M*,

Δ*M*_in_ *= M β*.

Therefore,

−Δ*M*_perp_ = Δ*M*_in_ *β*/2 = (Δ*M*_in_)^2^/ (2*M*). (S7)

Equation (S7) indicates that −Δ*M*_perp_ ($\propto$−Δ*ε_xy_*) is proportional to (Δ*M*_in_)^2^.

−


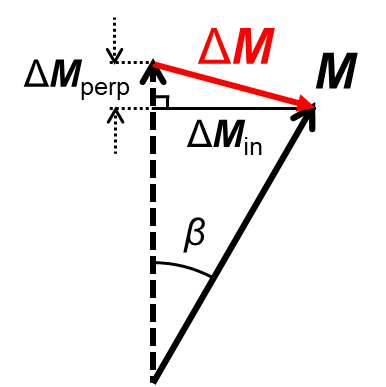


**Figure S5 | Derivation of the relationship between Δ*M*_perp_ and Δ*M*_in_.** The magnetization direction, the initial magnetization direction and the change in the magnetization direction are shown by the black dotted arrow, black solid arrow and red solid arrow, respectively.

**D. Landau-Lifshitz-Gilbert (LLG) simulation**

We performed the LLG simulation for GaMnAs using the well-known software OOMMF. The time evolution of $-\Delta$*M*_perp_ divided by the saturation magnetization (*M*_s_) is shown by red solid curve in Fig. S6a. *M*_perp_ is modulated by up to 0.001% in the LLG simulation. As a reference, the time evolution of $-\Delta\varepsilon_{xy}/\varepsilon_{xy}$ is also shown by the dark blue plots (same data as that shown in Fig. 3a in the manuscript) in Fig. S6b. The experimental $-\Delta\varepsilon_{xy}/\varepsilon_{xy}$-*t* curve shows that *M*_perp_ is modulated by up to 1%, which is three orders of magnitude larger than that calculated by the LLG-torque model. Hence, we concluded that the terahertz modulation of the magnetization is not caused by the magnetic field of the terahertz pulse.

**
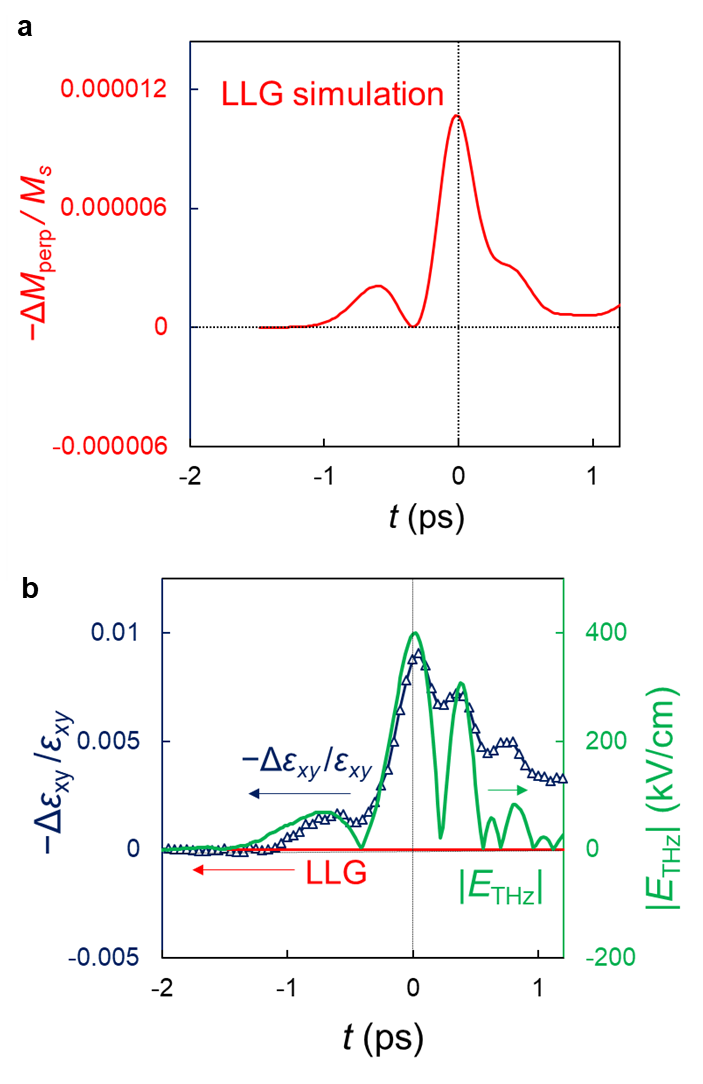
**

**Figure S6 | LLG simulation of the time evolution of the magnetization in the GaMnAs thin film using the micro-magnetics simulation package OOMMF. a**, Perpendicular magnetization dynamics derived from the LLG simulation for GaMnAs. **b**, Comparison between our experimental results shown by the dark blue plots (same data as that shown in Fig. 3a in the manuscript) and the LLG simulation shown by the red solid curve. |*E*_THz_| is shown by the green solid curve.

**References**

1. S. Novelli, F., Fausti, D., Giusti, F., Parmigiani, F. & Hoffmann, M. Mixed regime of light-matter interaction revealed by phase sensitive measurements of the dynamical Franz-Keldysh effect. *Sci. Rep.* **3**, 1227 (2013). [↑](#endnote-ref-1)
2. S. Kahn, F. J., Pershan, P. S. & Remeika, J. P. Ultraviolet magneto-optical properties of single-crystal orthoferrites, garnets, and other ferric oxide compounds. *Phys. Rev.* **186**, 891 (1969). [↑](#endnote-ref-2)
3. S. Matsuda, T. & Munekata, H. Mechanism of photoexcited precession of magnetization in (Ga,Mn)As on the basis of time-resolved spectroscopy, *Phys. Rev. B* **93**, 075202 (2016). [↑](#endnote-ref-3)
